# Inhaler technique: facts and fantasies. A view from the Aerosol Drug Management Improvement Team (ADMIT)

**DOI:** 10.1038/npjpcrm.2016.17

**Published:** 2016-04-21

**Authors:** Mark L Levy, P N R Dekhuijzen, P J Barnes, M Broeders, C J Corrigan, B L Chawes, L Corbetta, J C Dubus, Th Hausen, F Lavorini, N Roche, J Sanchis, Omar S Usmani, J Viejo, W Vincken, Th Voshaar, G K Crompton, Soren Pedersen

**Affiliations:** 1 General Practitioner and Respiratory Lead, Harrow, London, UK; 2 Radboud University Nijmegen Medical Centre, Nijmegen, The Netherlands; 3 National Heart and Lung Institute, Imperial College London, London, UK; 4 University Medical Centre Nijmegen, Nijmegen, The Netherlands; 5 Department of Respiratory Medicine and Allergy, King’s College London School of Medicine, London, UK; 6 COPSAC, Copenhagen Prospective Studies on Asthma in Childhood, Herlev and Gentofte Hospital, University of Copenhagen, Copenhagen, Denmark; 7 Department of Experimental and Clinical Medicine, University of Florence, Florence, Italy; 8 Unité de Medicine Infantile, Marseille, France; 9 Essen, Germany; 10 Service de Pneumologie et Soins Intensifs Respiratoires, Groupe Hospitalier Cochin, Université Paris-Descartes, Paris, France; 11 Departament de Pneumologia, Hospital de la Santa Creuide Sant Pau, Universitat Autónoma de Barcelona, Barcelona, Spain; 12 NIHR Career Development Fellow, National Heart and Lung Institute (NHLI), Imperial College London, UK; 13 Royal Brompton Hospital, London, UK; 14 Hospital General Yagüe de Burgos, Spain; 15 Universitair Ziekenhuis Brussel, Vrije Universiteit Brussel, Dienst Pneumologie, Brussels, Belgium; 16 Krankenhaus Bethanien, Moers, Germany; 17 Edinburgh, UK; 18 Pediatric Research Unit, University of Southern Denmark, Kolding Hospital, Kolding, Denmark

## Abstract

Health professionals tasked with advising patients with asthma and chronic obstructive pulmonary disease (COPD) how to use inhaler devices properly and what to do about unwanted effects will be aware of a variety of commonly held precepts. The evidence for many of these is, however, lacking or old and therefore in need of re-examination. Few would disagree that facilitating and encouraging regular and proper use of inhaler devices for the treatment of asthma and COPD is critical for successful outcomes. It seems logical that the abandonment of unnecessary or ill-founded practices forms an integral part of this process: the use of inhalers is bewildering enough, particularly with regular introduction of new drugs, devices and ancillary equipment, without unnecessary and pointless adages. We review the evidence, or lack thereof, underlying ten items of inhaler ‘lore’ commonly passed on by health professionals to each other and thence to patients. The exercise is intended as a pragmatic, evidence-informed review by a group of clinicians with appropriate experience. It is not intended to be an exhaustive review of the literature; rather, we aim to stimulate debate, and to encourage researchers to challenge some of these ideas and to provide new, updated evidence on which to base relevant, meaningful advice in the future. The discussion on each item is followed by a formal, expert opinion by members of the ADMIT Working Group.

## Introduction

*‘It ain't what you don't know that gets you into trouble.* *It's what you know for sure that just ain't so’.* *Mark Twain*

Inhaled medication is essential for the treatment of patients with asthma and chronic obstructive pulmonary disease (COPD), and incorrect use has well-demonstrated detrimental implications for patients.^1^ The effects of these medications depend, among other factors, upon the amount of the drug deposited in the lower airways. This is determined by the patient’s inhalation technique, which is influenced by characteristics of the individual inhaler devices and the medication therein.

There are two principal types of inhalation device: pressurised metred dose inhalers (pMDIs) and dry powder inhalers (DPI). Although advice about how to use these devices properly originates from many sources, it is typically provided by the patient’s health professionals, including pharmacists. This insular arrangement may result in propagation of local habits and beliefs, which may not be evidence based.

The purpose of this pragmatic, evidence-informed review is to consider the evidence, or lack thereof, underlying ten common ‘inhaler lore’ beliefs, selected on the basis of the combined clinical experience and research expertise of the authors.

The authors sourced evidence from their own bibliographical databases supplemented by a targeted Pubmed search using the following terminology: (Inhaler or DPI or ‘Dry Powder Inhaler’ or pMDI or ‘pressurised metred dose inhaler’) AND (‘inhalation therapy’ or ‘different inhalers’ or ‘mixed inhalers’ or ‘simultaneous use’ or ‘concomitant use’ or prime or priming or exhale or exhalation or hold or ‘breath hold’ or shake or shaken or rinse or rinsing or ‘mouth rinse’ or ‘mouth rinsing’ or spacer or empty or emptied or caries or ‘dental caries’ or dysphonia or hoarseness or thrush or candida or candidiasis or outcome or ‘clinical outcome’ or prefer or preference or oropharyngeal candidiasis or dysphonia or switch or single or multiple or tidal).

The 10 issues listed are not intended to be hierarchical or all-inclusive but do, in our view, cover the major issues related to current inhaler prescribing. Concluding statements follow the discussion on each topic and represent the consensus view of the panel.

## Beliefs, supporting data and panel conclusions

### 1. pMDI inhaler devices should be shaken

When chlorofluorocarbon (CFC) propellants were used in pMDIs, the active drug was typically included as a micronised suspension and not as a solution. As a result, failure to shake the device either before use or between successive dosages resulted in improper dispersion or ‘settling’ of the suspension in the propellant, which was clearly shown in studies to reduce the delivery of both β_2_-agonists and corticosteroids by up to 50%.^2,3^ Consequently, with the old CFC-driven pMDI devices, shaking the device before each and every dosage was critically important.

Modern pMDIs now universally contain hydrofluoroalkane (HFA) instead of CFC.^4^ In many, but in not all, of these devices, the active drugs are in true solution^5^ and thus shaking is unnecessary. It is the responsibility of the prescribing physician and dispensing pharmacists to determine whether or not any particular device contains drug in micronised form or in true solution when advising patients on the importance of shaking inhalers before use.

Pending clarification from the aerosol scientists and device manufacturers, we recommend shaking in the interim. While failure to shake some devices may reduce drug delivery, so far there have been no studies demonstrating any clinical consequences.

### Conclusions

1. The use of HFA-propelled drugs in true solution in pMDIs removes the necessity to shake the device.
2. Because not all pMDI devices contain drugs in true solution but in micronised form, advising shaking of the device before each and every dosage is the best default position where doubt exists.
3. DPI devices should not be shaken or inverted after priming, and prescribers should make patients aware of this.
4. Future studies are needed to clarify the clinical consequences, if any, of failure to shake relevant pMDI devices before each dosage and of DPI shaking before or after priming.

### 2. Spacer design and construction make a difference

Aside from removing the problem of co-ordinating activation of pMDIs with inhalation, the use of a spacer augments the time between expulsion of the aerosolised particles from the device and their inhalation. This serves two particular functions. First, it facilitates evaporation of the particles to a size capable of penetrating through the entire bronchial tree as far as the alveoli (i.e., with mass median diameter less than ~5 microns: particles emitted directly from a pMDI device are initially much larger). Second, it facilitates slow inhalation of the evaporated particles into the lungs, which is essential if they are to negotiate beyond the right-angle bend between horizontal emission from the spacer device and the vertical passage through the larynx. It has been shown that several varieties of commercially available spacer devices increase the proportion of particles emitted from a typical HFA-propelled pMDI device.^6^

Most spacer devices are constructed of plastic, which may accumulate electrostatic charge, which in turn attracts the aerosol particles, making them unavailable for inhalation. Several studies demonstrating this effect show that it is reduced by ‘priming’ of the device either by regular use or by spraying extra doses of the medication into the spacer before use (although this is wasteful).^7–10^ Regular detergent washing and drip-drying or air-drying (rather than wiping or rubbing, which adds to surface charge) of spacers is also helpful.^7,10^ Some spacers are constructed of non-electrostatic material. For example, the Nebuchamber (also known as the NES-spacer) is made of lightweight metal, which was shown not to require priming,^10,11^ and it has been suggested to improve total deposition of budesonide compared with plastic spacer devices.^9^ Similarly, the Aerochamber Max is a small-volume device constructed from a charge-dissipative polymer, which has been shown to emit a significantly greater proportion of fine mass particles (drug particles <5 microns in diameter), with either a 2- or 5 -s delay, compared with spacers made from non-conducting materials, even with pre-washing.^12^

It is important for patients to understand that, when using a spacer, inhalation should commence promptly:^13^ that is, within 3 s of actuating the pMDI. This is because aerosolised drug particles remain suspended in the the spacer for less than 10 s.^14^

Despite these observations, few, if any, studies have demonstrated clear benefits of spacer handling or design in clinical practice. One study of 64 young children found no difference in the bronchodilatation caused by salbutamol delivered by an HFA-propelled pMDI through a brand new Babyhaler, a detergent-coated, reduced static Babyhaler or a metal NES-spacer.^15^ In another study on young children treated with fluticasone propionate administered with an HFA-propelled pMDI, anti-static treatment of the valve-holding chamber and mask, which was being used to deliver the drug, resulted in increased lung bioavailability, but the possible beneficial clinical consequences of this were not examined.^16^

Taking multiple tidal breaths from a large-volume spacer may be more practical than taking single deep breaths, especially for children, and this appears to be effective at least in terms of bronchodilation.^17^ The number of breaths required to empty the spacer obviously depends on the size of both the device and the patient. Bisgaard^18^ recommends 10 breaths in infants, 5 breaths in toddlers and 2 deep inhalations in older children and adults.

Although it is clear therefore that individual spacer devices may vary in their emission of fine mass particles and their propensity to accumulate static charge, there is a paucity of clinical evidence on which to base recommendations about using one device over another.

### Conclusions

1. Spacer devices should not be considered interchangeable, as:
  - Metal spacers do not need priming.
  - Plastic spacers may become more efficient after priming, but the clinical implications of this are unclear and the procedure is wasteful of medication.
2. Detergent-washing and drip-drying/air-drying (rather than wiping or rubbing, which adds to the surface charge) of spacers is recommended on a regular basis, although to our knowledge this has not been demonstrated to improve asthma control. Nevertheless, it seems sensible to adhere to manufacturers’ advice regarding washing and routine replacement of spacer devices, and clearly they should be replaced if irretrievably soiled or damaged.
3. Studies are required to investigate the possible clinical consequences, if any, of using different spacer devices with or without measures to reduce static charge.

### 3. Breath-holding after inhalation is clinically beneficial

Inhaled particles exert their therapeutic effects after making contact with the surface of the bronchial or alveolar epithelium and delivering the drugs they contain to their receptors on cells situated therein. As this contact is initiated by random collision of the particles with the epithelial surface during Brownian motion,^19^ it would appear intuitive to hypothesise that exhalation soon after inhalation, particularly with fine particles before they interact with the epithelium, could reduce clinical efficacy of the drug. It is very difficult to address this hypothesis because many other aspects of inhaler technique, such as the speed and extent of inhalation, can also influence particle deposition. Furthermore, it is also very difficult to detect small changes in clinical effects in the short term that may result from breath-holding.

One study^20^ using radiolabelled Teflon particles with a mass median aerodynamic diameter of 3.2 microns found that a 10- s compared with a 4-s breath-hold significantly increased lung deposition of the particles, but only if (i) inspiration was initiated at 50–80% of vital capacity, which is not how inhalers are generally taken, and (ii) inspiration was slow (25 l m^−1^, as is generally recommended routinely). There was no measurable effect when inhalation was initiated at 20% of vital capacity (the more usual situation when using an inhaler routinely) or when the inspiratory flow rate was high (approximately 80 litres per minute).^20^ Similar findings were reported in another study.^21^ A pharmacokinetic study using carefully standardised inhalation manoeuvres showed significantly greater bioavailability of salbutamol absorbed from the airways following a 10-s breath-hold compared with no breath-hold.^22^

Other studies have addressed this hypothesis by using the clinical bronchodilator response to an inhaled β_2_-agonist as an end point.^23–28^ None of these demonstrated any significant difference in the bronchodilatation induced by a 10-s as compared with a 4-s breath-hold or no breath-hold.

A recent review noted variation in advice provided about breath-holding in a group of patient-orientated checklists produced by the manufacturers of various inhalation devices: in particular, one DPI manufacturer made no reference to the necessity for breath-holding at all. In view of this, the authors suggested a pragmatic approach by advising patients to hold their breath for at least 5 s.^29^

In summary, the conclusion that breath-holding significantly influences the clinical effects of inhaled bronchodilators must remain tentative. A caveat is that all of the studies cited above were performed with now obsolete, relatively large particle CFC propellant-driven pMDIs. To our knowledge, there are no studies of the effects of breath-holding time on wanted or unwanted effects of DPIs, spacer devices or fine-particle pMDIs delivering bronchodilators or inhaled corticosteroids. At least in theory, the effect of breath-holding time may be more critical with fine-particle pMDIs, as one might hypothesise that these are likely to require a more protracted period to settle in the airways through Brownian motion, but so far as we are aware there are no relevant studies.

### Conclusions

1. Breath-holding after inhalation from a pMDI may increase deposition of the inhaled drug in the airways, but no studies, to our knowledge, have demonstrated improved bronchodilatation or any long-term therapeutic consequences as a result.
2. As it is possible that breath-holding may be beneficial when using inhaler devices with an otherwise perfect technique, a pragmatic approach, until further evidence becomes available, is to advise all patients to breath-hold for at least 5 s.
3. Future studies should investigate the clinical importance of breath-holding when using dry powder inhalers, spacer devices and fine-particle pMDIs.

### 4. Rinsing of the mouth after using an inhaler is clinically beneficial

Patients are often advised to ‘rinse and spit’ or gargle after using an inhaler device, particularly one containing corticosteroid. Intuitively, this might be expected to decrease local oropharyngeal adverse effects of inhaled corticosteroids, especially oral candidiasis (thrush), although not dysphonia because the procedure cannot possibly irrigate the larynx.

A meta-analysis of 23 studies^30^ showed a fivefold prevalence of oral candidiasis in patients taking inhaled corticosteroids with a pMDI device and a threefold increase in those using DPIs, although there is wide variation between studies both in prevalence of clinical signs and symptoms and the correlation of these with the ability to culture yeasts from throat swabs.^31,32^ A large questionnaire study of almost 900 patients suggested a significant, threefold reduction of subjective oral symptoms by mouth washing after using inhalers, but curiously this was observed only in females using DPIs (and not pMDIs).^33^

Two studies^34,35^ showed that mouth washing after using inhalers reduced the amount of residual drug remaining in the oropharynx, but the possible clinical consequences of this observation were not assessed. A further study suggested that ‘throat washing’ reduced colonisation of the oropharynx with *Candida* species in patients inhaling fluticasone propionate using a dry powder inhaler.^36^ Yokoyama *et al.* ^37^ showed that different types of mouth rinsing may be important, with rinsing and gargling more effective than rinsing alone for removing oropharyngeal fluticasone propionate deposited after using a DPI. An additional potential benefit of mouth rinsing after inhaling corticosteroids might be reduced deposition in the gut and thus reduced systemic exposure. This is most likely to be relevant for beclomethasone dipropionate, which has some oral bioavailability, but unlikely to be relevant with other commonly used inhaled corticosteroids, such as fluticasone propionate and budesonide, as their oral bioavailability is so low. There do not appear to be any specific studies on the effects of mouth rinsing on systemic absorption of any inhaled corticosteroid.

Some studies suggest that dental caries are more frequent in asthmatic than in non-asthmatic children,^38^ and one study^39^ reported that caries were somewhat less common in asthmatic children undertaking regular mouth rinsing, although this difference did not achieve statistical significance. In this study, the lactose content of the filler in some of the DPI devices did not emerge as a particular risk factor for caries. In another case–control study of asthmatic patients aged 10–45 years using DPI-delivered inhaled corticosteroids, there was no significant difference in the incidence of dental caries in the asthmatics compared with matched controls, although there was some correlation within the asthmatic group between the incidence of caries and the frequency of DPI use. Whether this reflects possible effects of the drug or the filler, or both, is unknown.^40^

Aside from this tentative, largely indirect evidence, there is no clear evidence that dental caries are caused by inhaled anti-asthma drugs. It has been hypothesised that dental caries may be an unwanted effect of inhaled beta-2-agonist drugs reducing oral pH.^41^ If this were the case, then clinical studies of the effects of mouth rinsing on the incidence of caries might be confounded by the fact that, in general, patients are advised to mouth-rinse after inhaling corticosteroids but not ‘relievers’.

In summary, therefore, as far as we are aware the evidence for any recommendation for regular mouth rinsing following the use of inhaled corticosteroids is entirely empirical.^42^ One might speculate that, with the increasing use of pMDI devices with HFA propellants, which deliver drugs in true solution, and the increased use of pro-drugs such as ciclesonide^43^ and beclomethasone dipropionate,^44^ which are metabolised to be active in the lower respiratory tract rather than the mouth, this problem, if it exists, may become less prevalent.

### Conclusions

1. Although it reduces residual deposition of inhaled anti-asthma drugs, in the oropharynx, there is no clear evidence that mouth rinsing reduces oral thrush or dental caries or influences the systemic bioavailability of the drugs.
2. There are some data to suggest that children should be advised to mouth-rinse after inhaling β_2_-agonists to reduce dental caries, but this requires confirmation.
3. Future studies should investigate how β_2_-agonists and corticosteroids inhaled from pMDIs and DPIs affect the oral microbiome and whether or not mouth rinsing has clinical benefits in preventing thrush and caries in children and adults.

### 5. Moisture and dry powder inhalers (DPIs)

The release of respirable drug particles from DPIs requires considerable force and at a relatively high flow rate to disperse the powder and/or de-aggregate the particles to a respirable size (<5 microns).^45^ This effort is required to overcome inter-particular forces including Van der Waals forces and electrostatic charge, more manifest with smaller particles.^46^

All powder formulations are sensitive to humidity, and dispersion of fine particles is particularly impaired by moisture. *In vitro* studies have demonstrated reduction in the fine-particle fraction of dry powder aerosols with increasing relative humidity^46–48^ and that some products are more susceptible to ambient humidity than others.^49,50^ There is some evidence that devices employing a reservoir to store the dry powder are more susceptible to humidity than those in which the powder is stored in individual sealed blisters or capsules,^47,51^ although even the latter are somewhat sensitive to moisture. In addition to ambient moisture, drugs delivered by DPI devices are also vulnerable to wetting by inappropriate breathing into the device, which causes condensation^52^ and a reduction in the available inhaled fine-particle fraction of the drug.^48^

Remarkably, despite this relative wealth of data, we are aware of no studies whatsoever reporting the effects of ambient humidity on any clinical outcome of DPI therapy. Nevertheless, it would seem sensible to instruct patients to store DPI devices in a dry environment away from obvious sources of moisture, and not to exhale or blow into them at any time.

### Conclusions

1. Dry powder devices should be stored in a dry environment, as humidity reduces the dispersal properties of DPIs.
2. Patients should be instructed not to exhale or blow into their inhaler device, as this causes condensation and humidity.
3. Future studies should investigate whether ambient humidity has clinical consequences in patients using β_2_-agonists and corticosteroids inhaled from different DPIs.

### 6. Patients can determine when their pMDI is empty

Clinical risk for patients who continue to use an empty inhaler is obvious. There is a self-evident need for manufacturers and prescribers to ensure that patients know how to keep track of the dosages remaining in their inhalers.

While some pMDIs are provided with a dosage counter, many are not. Without one it is very difficult to determine when the device is empty. Some patients are instructed to keep track^53,54^ of the dosages they use, but this can be cumbersome and unreliable, and many patients are in any case not aware of the specified maximum number of actuations listed by the manufacturer.^55,56^ In practice, a significant number of patients discover that their inhalers are empty too late to avoid emergency hospital attendance.^55^ Further, the absence of a counter may conversely result in inappropriate early renewal of prescriptions and wastage. In one study, more than 50% of 500 patients interviewed stated that they refilled their prescriptions earlier than recommended by national guidelines.^55^

This problem has long been recognised. ‘Floating’ of pMDIs in water has been recommended^53,56–58^ as a means of determining how full the device is, but this practice is inaccurate,^59^ may be product specific^54,59^ and in over 25% of cases in one study^60^ it resulted in water entering the device and obstructing the valves. Consequently, this approach is clearly inadmissable. Currently, therefore, there are no reliable means by which patients can monitor the number of dosages remaining in their pMDIs unless the device has a dosage counter.

### Conclusions

1. Patients cannot reliably determine the remaining dosages in a pMDI without a counter.
2. Prescribers should favour a device with a dose counter.
3. If prescribing a device without a counter, ensure that the patient is aware of the need to keep track of remaining medication. A practical but work-intensive solution is to keep count of the dosages used, and to advise the patient to maintain a spare device available at all times.
4. Future studies are needed to examine means to determine when a pMDI without a counter is empty.

### 7. The use of a single design of inhaler to deliver different inhaled drugs improves clinical outcomes

As pMDI and DPI devices require very different techniques for optimal performance, it is reasonable to hypothesise that handling errors and suboptimal drug delivery will be multiplied when a patient has to use a range of different devices. This has been verified in several studies showing that simultaneous use of different types of inhaler device, particularly a mixture of pMDI and DPI devices, is clearly predictive of increased errors in inhalation.^61,62^

Asking patients to mix inhaler devices may also adversely affect their compliance with therapy. In a retrospective observational study of over 11,000 COPD patients, multiple-inhaler users were less likely to adhere and significantly more likely to discontinue therapy altogether than single-inhaler users.^63^

In contrast, some studies suggest that mixing inhalation strategies can improve clinical outcomes. For example, a study on 126 patients with COPD treated with salbutamol and ipratropium^64^ found that health-related quality of life and symptom scores improved significantly more when using a combination of nebuliser therapy morning and night along with a pMDI device in the afternoon and evening rather than using either of these devices alone four times daily. The relative advantages and disadvantages of using similar or dissimilar inhaler devices to deliver both preventer and reliever therapy remains a subject of lively debate.^65,66^

Although it would seem logical, wherever possible, to prescribe drugs in identical devices or, if this is not possible, to confine therapy to either pMDI or DPI devices rather than a mixture of the two, evidence is lacking at least in terms of long-term clinical outcomes in populations.

### Conclusions

1. Although there is evidence that asking patients to use inhalers of a single type reduces errors and may improve compliance, there is a paucity of evidence showing corresponding favourable clinical outcomes.
2. In the light of currently available evidence, it would seem reasonable to restrict regular (preventer) inhaled medication to a single type of device ( pMDIs or DPIs) whenever possible.
3. Nevertheless, future studies are warranted to examine whether using particular combinations of therapeutic strategies in specific situations when treating patients with asthma and COPD may be clinically beneficial.

### 8. Patients who use an inhaler device they prefer have better outcomes

The choice of devices when prescribing inhaled medication for patients with asthma and COPD is influenced by many factors including the patient’s preference and ability to use the device, the availability of the drug in the preferred device and sometimes the cost of the therapy and potential for reimbursement.^67^ An observational study^68^ investigating the relationship between patients’ satisfaction with their inhaler devices, their compliance with therapy and the influence of these factors on health and self-reported outcomes tentatively concluded that patient satisfaction with inhaler devices influenced the attainment of asthma treatment goals,^69^ largely through improving compliance. It is difficult to assess in practice precisely what influences prescribing of particular inhaler devices. For example, although it is well known that subsets of patients find pMDIs difficult to use correctly^1,70–72^ they are still widely prescribed; whether this reflects patient or health-care professional preference is not clear. When helping patients choose inhalation devices, they prefer some objective input may be obtained by using appropriate questionnaires such as the satisfaction with asthma treatment questionnaire and others.^73–75^ These tools may also be used to highlight patients who particularly dislike their existing device, suggesting the need for a change. Although patient preference may be ideal in deciding which device to prescribe, prescribers should ensure that patients are able to use the prescribed device correctly.^76^

### Conclusions

1. There is limited evidence supporting the hypothesis that asthmatic patients’ satisfaction with their inhaler device is associated with improved disease control.
2. This satisfaction, or otherwise, should be assessed along with other techniques, at asthma reviews, and the device should be changed if appropriate.
3. Future studies should investigate, for example, using established questionnaires, whether patients who are satisfied with their devices have better outcomes.

### 9. Dysphonia is caused by particular inhaler devices and changing the device may relieve it

Dysphonia is a recognised unwanted local adverse effect of inhaled corticosteroid therapy. In response to a patient’s complaint of dysphonia, the attending health professional will often be tempted to consider changing the inhalation device. There is in fact little clinical evidence to justify or guide such a strategy. A recent review^43^ concluded that dysphonia may be improved by changing the inhaler device, or by reducing the amount and/or frequency of inhaled corticosteroid therapy, or changing the inhaled corticosteroid to ciclesonide (a small particle, true solution pro-drug not active in the upper airways).

To address these issues adequately, studies are required in which the same corticosteroid at the same dosage is administered to patients with various devices, preferably in a crossover design, but to our knowledge such studies have not been published. One study investigated whether *ad hoc* changing of the device used to deliver inhaled corticosteroid therapy has any demonstrable effect on the voice.^77^ Patients reporting dysphonia while taking an inhaled corticosteroid with a pMDI device alone were randomised either to continue with a pMDI device with a Nebuhaler spacer or to switch to an identical dosage of corticosteroid delivered using a Turbohaler. Twelve weeks after switching, there were no detectable differences in voice laboratory data, laryngoscopic evidence of disordered glottic closure and symptom diary data between the two groups. Gross laryngoscopic appearances of the vocal cords were normal in almost half of the patients in the study. Vocal cord bowing was rarely observed. Glottic closure was detectably altered in 9 patients during the study period, but this did not correlate with reported problems of vocalisation. Four weeks after switching, 40% of the patients using the Turbohaler and 8% of those using the pMDI and Nebuhaler scored their voice status as significantly improved (*P*<0.02 for a between group difference), but there was no significant difference between the groups at 12 weeks (Turbohaler 52% improvement, Nebuhaler 23% improvement, *P*=0.08 for between-group difference).

### Conclusions

1. Dysphonia is a recognised side effect of inhaled corticosteroid therapy, but its relationship to the use of particular inhaler devices, if any, is unclear.
2. There is a paucity of published evidence that changing inhalation devices used to deliver topical corticosteroids can relieve dysphonia.
3. Future studies are warranted to examine new strategies to alleviate complaints of dysphonia, and to clarify the ability of corticosteroid pro-drugs such as ciclesonide to reduce its incidence.

### 10. Regular and proper use of inhaler devices improves asthma outcomes

There is evidence in the literature^1,78,79^ linking incorrect inhaler technique with poor treatment outcomes in asthma and COPD such as treatment failure, unnecessary escalation of therapy and increased exacerbations with unplanned use of medical services and hospitalisation. These studies justify the statement, often emphasised in guidelines, that if patients use their inhalers correctly their asthma is likely to be better controlled.^80–83^

Separately, a number of carefully performed observational studies^84–86^ have demonstrated that patients who follow their prescribed treatment plan and adhere closely to prescribed therapeutic regimens show a number of favourable outcomes including fewer symptoms and exacerbations, reduced use of rescue medication and even reduced loss of lung function over the years.^87,88^ Conversely, poor adherence is associated with increased morbidity and poorer clinical outcomes.^89,90^

It is tempting to assume that poor adherence to therapy and poor inhaler technique go hand in hand in individual patients, but so far there are no studies that clearly demonstrate this. Consequently, it seems wise to address both issues separately during reviews of asthma management. Although they do not necessarily go hand in hand, careful instruction on inhaler technique may confer the additional benefit of improving adherence to therapy where it is lacking, and better outcomes.^91^

### Conclusions

1. Both incorrect inhaler technique and irregular usage of inhaler medication are common causes of poor asthma control.
2. Both must be diligently and regularly reviewed.
3. Future studies should investigate whether poor inhaler technique and poor adherence coexist in the same patients, and how they interact to influence treatment outcomes.

## Afterword

We have presented this summary of the literature and our own conclusions in order to stimulate debate about perfecting the subtle and essential art of successful inhaler therapy. Although national and international asthma and COPD guidelines address overarching issues such as the necessity to take inhaled medications for obstructive airways disease, they tend not to address the practical details. We felt it timely to take a critical view of the commonly held beliefs that have become accepted truths of inhaler ‘lore’ in the eyes of many health professionals. Hopefully, we have raised sufficient doubt in the minds of clinicians to stimulate further research on the practical aspects of inhaler prescribing.
