# Inhaler technique: facts and fantasies. A view from the Aerosol Drug Management Improvement Team (ADMIT)

**DOI:** 10.1038/npjpcrm.2016.28

**Published:** 2016-05-26

**Authors:** Mark L Levy, PNR Dekhuijzen, PJ Barnes, M Broeders, CJ Corrigan, BL Chawes, L Corbetta, JC Dubus, Th Hausen, F Lavorini, N Roche, J Sanchis, Omar S Usmani, J Viejo, W Vincken, Th Voshaar, GK Crompton, Soren Pedersen

**Correction to:** *npj Primary Care Respiratory Medicine* (2016) **26,** 16017; doi:10.1038/npjpcrm.2016.17; published online 21 April 2016

Since the online publication of the above article, it has been noted that references 87 and 88 were described as observational studies, when in fact they were randomised controlled trials.

**On page 5, section 10 of the paper, the following text:**

Separately, a number of carefully performed observational studies^84,85,86^ have demonstrated that patients who follow their prescribed treatment plan and adhere closely to prescribed therapeutic regimens show a number of favourable outcomes including fewer symptoms and exacerbations, reduced use of rescue medication and even reduced loss of lung function over the years.^87,88^ Conversely, poor adherence is associated with increased morbidity and poorer clinical outcomes.^89,90^

**Has been replaced by:**

Separately, a number of carefully performed observational studies^84^ ^–86^ have demonstrated that patients who follow their prescribed treatment plan and adhere closely to prescribed therapeutic regimens show a number of favourable outcomes including fewer symptoms and exacerbations, and reduced use of rescue medication; and randomised controlled trials have shown reduced loss of lung function and mortality in patients with COPD,^87^ and improved lung function and quality of life in people with asthma.^88^ Conversely, poor adherence is associated with increased morbidity and poorer clinical outcomes.^89,90^

The errors have been corrected in the PDF and HTML versions of the article.

